# High-Accuracy Long-Read Sequencing of *Mycobacterium tuberculosis* PSNK363 Isolated From the Democratic People's Republic of Korea

**DOI:** 10.1155/cjid/2234550

**Published:** 2025-02-11

**Authors:** Thi-Binh Dang, Nackmoon Sung, Kyunghyun Lim, Soyoung Lee, Jaehyun Jeon, Sanghoon Jheon

**Affiliations:** ^1^Department of Thoracic and Cardiovascular Surgery, Seoul National University Bundang Hospital, Seongnam-si, Gyeonggi-do, Republic of Korea; ^2^Department of Thoracic and Cardiovascular Surgery, College of Medicine, Seoul National University, Seoul, Republic of Korea; ^3^Healthcare Division, MediQstar Co., Ltd., Seoul, Republic of Korea; ^4^Department of Medical Device Development, College of Medicine, Seoul National University, Seoul, Republic of Korea

**Keywords:** Democratic People's Republic of Korea, genomic, long-read sequencing, *Mycobacterium tuberculosis*, tuberculosis

## Abstract

Long-read sequencing is a valuable technique for high-precision genome analysis. Despite the widespread use of the *Mycobacterium tuberculosis* H37Rv genome sequence as a reference for genetic variation analysis, its suitability for comparing clinical strains is limited. Therefore, we constructed the first known whole genome of a clinical *M. tuberculosis* strain, PSNK363, isolated from the Democratic People's Republic of Korea, using high-quality high-fidelity (HiFi) read sequencing and compared its genetic variations to those of H37Rv. PSNK363 was cultured to obtain genomic DNA, which was subjected to *de novo* whole-genome assembly using PacBio Sequel II with long-read HiFi sequencing. The sequences were compared to the reference genome H37Rv. HiFi long-read sequencing of *M. tuberculosis* PSNK363, with an accuracy of 99.99%, revealed a single circular chromosome of 4,422,110 bp, which is 10,578 bp longer than the H37Rv chromosome. The assembly had an average G + C content of 65.6%, 4079 protein-coding sequences, 53 tRNA genes, and 3 rRNA genes. Most genes (72.7%) were assigned as putative functions, whereas the remaining 27.3% were annotated as hypothetical. Comparison with H37Rv revealed a large inversion in the PSNK363 genome, which contains most of the deletion and insertion variants. *M. tuberculosis* PSNK363 had a longer genome sequence, more protein-coding genes, and a larger inversion region than H37Rv. High-accuracy whole-genome sequencing of PSNK363 holds the potential for enriching virulence databases and identifying informative loci for drug resistance analysis in *M. tuberculosis* isolates in the Democratic People's Republic of Korea.

## 1. Introduction

Tuberculosis (TB) is a serious public health problem in the Democratic People's Republic of Korea (DPRK), with an estimated incidence rate of 513 per 100,000 people for TB and 5200 cases of multidrug-resistant (MDR)-TB in 2022 [1]. This places the country among the 30 high-burden countries for TB and MDR-TB/rifampicin (RIF)-resistant (RR)-TB [1]. Despite the availability of diagnostic tests and drugs for MDR-TB treatment, the regimens are more expensive and arduous than those for drug-susceptible TB, resulting in high treatment failure and mortality rates. A study from sanatoria in the DPRK [2] indicated a considerable percentage of drug-resistant TB isolates, while other studies [3, 4] raised concerns regarding the need to scale up MDR-TB treatment in the DPRK. While favorable treatment outcomes in patients with MDR-TB have been observed in the DPRK [3], the high number of TB and MDR-TB cases owing to challenges in accessing appropriate treatment medications [4] poses a risk of further spread. Additionally, the factors driving the MDR-TB epidemic in the DPRK remain unclear, and data on *Mycobacterium tuberculosis* genome sequencing in the DPRK are insufficient and unpublished.

To address this genomic data gap on *M. tuberculosis* in the DPRK, we performed whole-genome sequencing of a clinical sample obtained from the Eugene Bell Foundation collection [2]. The foundation transferred sputum samples of patients in the DPRK to Masan National TB Hospital (Masan, South Korea) from 2007 to 2009 for repetitive sequence-based polymerase chain reaction (rep-PCR) genotyping using the DiversiLab® system (bioMerieux). Subsequently, the strains were stored and provided to Seoul National University Bundang Hospital (SNUBH) for genomic sequencing. Among 179 *M. tuberculosis* isolates studied in this collection, only 7 (3.9%) were confirmed to be susceptible to drugs using the conventional method with Löwenstein–Jensen (LJ) medium. *M. tuberculosis* PSNK363 was derived from these susceptible isolates and belongs to the most prevalent cluster identified through rep-PCR.

Long-read sequencing was recognized as the “Method of the Year 2022” [5] owing to its exceptional potential in genome, transcriptome, and epigenome analyses. Specifically, the PacBio long-read sequencing is known for its high accuracy, rendering it suitable for applications that require precise single nucleotide variant (SNV) detection [6], such as genetic analysis of *M. tuberculosis*. Hence, in the present study, we employed highly accurate long-read high-fidelity (HiFi) sequencing to construct a whole-genome sequence of the unique *M. tuberculosis* strain PSNK363 from the DPRK and performed a comparative analysis with H37Rv.

## 2. Materials and Methods

### 2.1. Selection of *M. tuberculosis* PSNK363

*M. tuberculosis* PSNK363 was obtained from a collection of 179 clinical *M. tuberculosis* isolates maintained as part of our previous study [2], stored at Masan National TB Hospital (South Korea), and transferred to SNUBH, South Korea, for whole-genome sequencing.

### 2.2. Ethics Approval

This study was performed in line with the guidelines of SNUBH and approved by the Institutional Review Board (approval number: X-2008-631–901) and Institutional Ethics Committee (approval number: IBC-2002-A-001-02) of SNUBH.

### 2.3. *M. tuberculosis* Culture, Drug Susceptibility Testing, and DNA Preparation

*M. tuberculosis* PSNK363 was aerobically cultured in Middlebrook 7H9 medium containing 0.05% (v/v) Tween 80% and 10% Middlebrook OADC growth supplement (Sigma-Aldrich, St. Louis, MO, USA) at 37°C.

The sensitivity of PSNK363 to RIF (40 μg/mL), isoniazid (0.2 μg/mL), ethambutol (2.0 μg/mL), levofloxacin (2.0 μg/mL), ethionamide (40 μg/mL), amikacin (30 μg/mL), and kanamycin (40 μg/mL) was confirmed via conventional methods using LJ medium. Pyrazinamide sensitivity was determined using pyrazinamidase assay with the Wayne method.

Genomic DNA was isolated from *M. tuberculosis* using the cetyltrimethylammonium bromide method. Briefly, the bacilli in suspension were heat-treated at 80°C for 30 min in a water bath. After centrifugation, the cell pellets were resuspended in 500 μL of Tris-EDTA buffer (0.01 M Tris–HCl and 0.001 M EDTA [pH 8.0]). Subsequently, the cells were treated with lysozyme (50 mg/mL) for 1 h at 37°C and then with 10% sodium dodecyl sulfate and proteinase K (10 mg/mL) for 20 min at 60°C. Thereafter, 200 μL of N-acetyl-N, N,N,-trimethyl ammonium bromide was added to approximately 500 μL of the lysed cell suspension. The mixture was briefly vortexed and incubated for 10 min at 60°C. An equal volume of chloroform-isoamyl alcohol (24:1, v/v) was added to the mixture, which was then centrifuged for 5 min; 0.6 volume of isopropanol was added to the supernatant to precipitate the DNA. After cooling for 30 min at 20°C, the DNA solution was centrifuged for 15 min, and the pellet was washed once with 70% ethanol. Finally, the air-dried pellet was redissolved in 50 μL of 0.1× Tris-EDTA buffer and stored at −20°C until further use.

### 2.4. DNA Quality Control

PSNK363 gDNA was quantified using the Qubit® fluorometer (Thermo Fisher Scientific, Waltham, MA, USA) with Broad Range dsDNA assay kits, and DNA purity was measured using the NanoDrop™ 2000/2000c spectrophotometer (Thermo Fisher Scientific). Fragment size was evaluated through agarose gel electrophoresis. PSNK363 gDNA adhered to the following criteria for sequencing: A260/280 ratio: 1.7–2.0; A260/230 ratio: 1.8–2.0; and DNA weight: > 5 μg/μL. The purified DNA was stored at 4°C prior to the preparation of sequencing libraries.

### 2.5. Whole-Genome Sequencing

**Figure 1:**
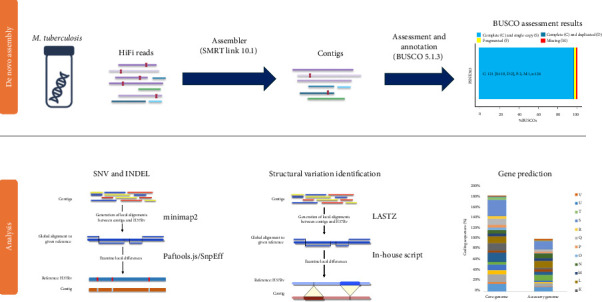
Workflow diagram of whole-genome sequencing of *Mycobacterium tuberculosis* PSNK363.

The *M. tuberculosis* PSNK363 genome was sequenced using PacBio Sequel II (Pacific Biosciences, Menlo Park, CA, USA) at Macrogen Inc. (Seoul, Republic of Korea) with a long-read HiFi library (Figure 1). The genomic DNA was fragmented using dsDNA fragmentase (New England Biolabs, Ipswich, MA, USA) to achieve the desired size (10–25 kb) for library construction. The resulting DNA fragments were then processed using the SMRTbell Express Template Prep Kit 2.02 (Illumina Inc., San Diego, CA, USA), following the manufacturer's instructions.

### 2.6. Bioinformatics and Data Analysis

#### 2.6.1. Genome Assembly

The generated PacBio sequencing reads (6,133,251 reads; total read length: 359,444,804,032 bp) and HiFi sequencing reads (1,621,177 reads; total read length: 23,913,685,936 bp) were assembled using the Phred/Phrap/Consed package and CLC Genomics Workbench v6.5 (CLC bio, Aarhus, Denmark). The resulting contigs from PacBio sequencing were scaffolded by sequencing the reads from the fosmid clones, and the gaps in the scaffolds were closed using PCR and Sanger sequencing. The contigs and Sanger sequence reads for gap closure were combined via manual curation using Phred/Phrap/Consed and CodonCode Aligner 3.7.1 (CodonCode Corp., Centerville, UT, USA). Genome assembly quality was assessed using Benchmarking Universal Single-Copy Orthologs (BUSCO 5.1.3) [7, 8]. A graphical circular representation of the genome was visualized using Circos v0.69-9 [9].

#### 2.6.2. SNVs and Small Insertion and Deletion (Indel) Analysis

HiFi reads were aligned to the reference H37Rv genome using minimap2 [10] to generate a Binary Alignment Map file, and Paftools.js was then applied to call the variants. Variant annotations were performed using SnpEff. The evolutionary genealogy of genes was functionally annotated using nonsupervised orthologous group (EggNOG) DB tools [11]. To generate the assembly used for gene prediction and annotation, Prokka 1.14.6 [12] was used with the genome assembly application. The predicted genes were functionally annotated using EggNOG v4.5, InterProScan 5.30–69.0 [13], and PSI-BLAST 2.10.0 [14].

#### 2.6.3. Structural Variant Analysis

The alignment results from the assembled contigs of PSNK363 and H37Rv were aligned using nucmer lastz. Subsequently, these alignments were processed using Structural Variants from MUMmer to identify structural variants, including deletions, insertions, and inversions.

## 3. Results

### 3.1. Genome Features of *M. tuberculosis* PSNK363 and Its Comparison With H37Rv

**Figure 2:**
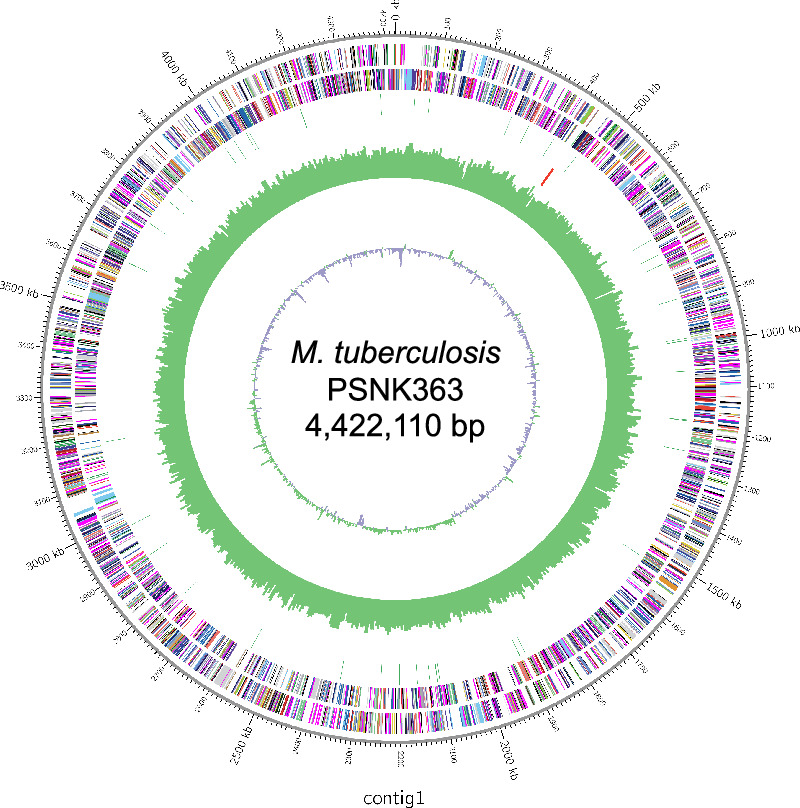
Circular map of *Mycobacterium tuberculosis* PSNK363 visualized using Circos [9]. Inner to outer: green and purple skew bars: GC content of coding sequence, green skew bar: SNVs relative to H37Rv, red and green bars: reverse and forward rRNAs, multicolor bars: reverse and forward coding sequences, and gray outside ring: assembled contig.

*M. tuberculosis* PSNK363 harbors a single circular chromosome of 4,422,110 bp (Figure 2). It possesses an average G + C content of 65.6% and comprises 4079 protein-coding sequences, 53 tRNA genes, and three rRNA genes (Table 1). The total length of HiFi reads is 1,621,177 bp, N50 contig length with HiFi reads is 15,079 bp, and the mean read length is 14,750 bp; thus, the HiFi accuracy was 99.99%. The genome of PSNK363 is 10,578 bp longer than that of H37Rv (4,411,532 bp).

### 3.2. Functional Classification of Orthologous Clusters

In this study, the annotation of protein sequences predicted by Prokka was refined using EggNOG. Overall, the annotation revealed the presence of 4146 genes, among which 4079 corresponded to coding sequences. Among the proteins coded by these coding sequences, 4006 were successfully matched to entries in the EggNOG database. Notably, 3946 proteins were associated with a single EggNOG category, whereas 60 proteins were linked to multiple EggNOG categories. However, 73 proteins revealed no significant similarity to known proteins in the EggNOG database.

Table 2 presents the EggNOG category distribution of functional annotation results and their frequencies. More than 27% (1112 proteins) of the proteins were categorized as having unknown functions. Additionally, a smaller proportion of proteins (3.5%; 146 proteins) was assigned to the EggNOG category related to translation, ribosomal structure, and biogenesis.

### 3.3. SNV Indels and Variant Calling

To identify SNVs and small indels, SNV calling was performed by comparing our assembly with the H37Rv genome. The assembled contig of PSNK363 and H37Rv revealed 2180 variants comprising 1582 SNVs, 321 insertions, and 277 deletions.

To interpret the effect of SNVs, the World Health Organization (WHO) catalog of mutations in *M. tuberculosis* [16] was used as a reference to search for variants appearing in the PSNK363 genome. As presented in Table 3, among the 16 identified variants, 12 sequences were described in the WHO catalog, and four sequences were not previously reported. Sequence variants that matched those in the WHO database [16] belonged to Group 4 and were not associated with resistance-interim, whereas Group 5 was not associated with resistance. Variants in PSNK363, including *eis*_c-3520A > C, *fgd1*_c.651C > G, *mmpL5*_c-2915A > G, and Rv2752c_c.489A > G, are not listed in the WHO catalog.

### 3.4. Structural Variants

For large variants (> 50 kb), the PSNK363 assembly contained 28 structural variants: 10 insertions, 15 deletions, and 3 inversions (Table 4).

**Figure 3:**
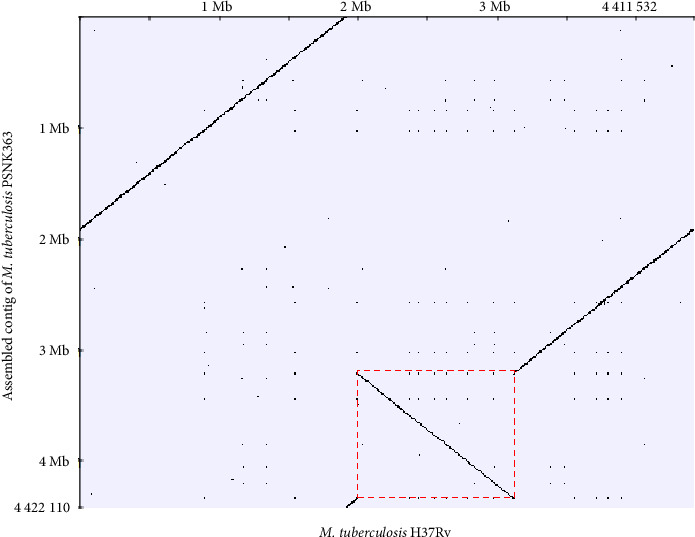
Large inversion detected in *M. tuberculosis* PSNK363. Genome dot plots of the PSNK363 assembled contig against *M. tuberculosis* H37Rv showing the presence of a large inversion (red dash area).

Upon comparison of the H37Rv and PSNK363 assemblies, an inversion sequence was observed in the syntenic dot plot (Figure 3). The inversion region of PSNK363 contains approximately 1,119,943 nucleotides at positions 3,217,011–4,336,953. The inversion sequence of PSNK363 results in two breakpoint regions in the genome sequence: a large breakpoint (Gap 1) at positions 4,336,953–4,345,124 and a smaller breakpoint (Gap 2) at positions 3,217,005–3,217,011. The inversion breakpoints were confirmed using PCR and resequenced using Sanger sequencing to assemble the PSNK363 genome (data not shown), which indicated the presence of an inversion in the PSNK363 chromosome.

## 4. Discussion

Whole-genome sequencing has revolutionized our understanding of drug resistance evolution, pathogenesis, and virulence in clinically relevant pathogens, such as *M. tuberculosis*. Single-molecule sequencing technology, particularly long-read sequencing, enables the generation of complete and closed microbial genome assemblies [17, 18]. This emerging technology can improve the accuracy of reference genome sequences and enable direct comparisons among different bacterial genomes without initially performing reference-based assembly [18, 19]. Although data on *M. tuberculosis* genomes from the DPRK are scarce [20], *M. tuberculosis* PSNK36 is the first strain with complete whole-genome isolated from patients with TB in a sanatorium in the DPRK using long-read sequencing.

Characterization of protein functions in *M. tuberculosis* is crucial for determining its pathogenicity, antibiotic resistance, and virulence. Our findings revealed that the functional annotation of PSNK363 is primarily associated with transcription (K); lipid transport and metabolism (I); replication, recombination, and repair (L); amino acid transport and metabolism (E); and energy production and conversion (C). These findings align with those of previous studies, which demonstrated a high percentage of genes involved in these groups across various *M. tuberculosis* populations [21, 22]. These findings suggest that these genes are typically conserved to ensure *M. tuberculosis* interactions with its host, particularly during mycobacterial persistence when host pathogens compete for nutrients and evade immune recognition [21]. Additionally, we classified SNVs related to resistance-conferring mutations of PSNK363 using the WHO mutation catalog [16]. We identified unique variants in PSNK363, including *eis* (c-3520A > C), *fgd1* (c.651C > G), *mmpL5* (c-2915A > G), and Rv2752c (c.489A > G), which were associated with susceptibility to certain genomic variants via a phenotypic method.

Gene inversion is a common phenomenon in bacterial evolution and can promote increased virulence and drug resistance in various pathogens [23, 24]. Large inversion events have been observed in some isolates belonging to the Beijing family [25–27] and KZN strains [28]. Our analysis revealed an inversion in the assembly of PSNK363, and notably, most indel events occurred around the inversion region. This finding suggests that inversions generate adaptive mutations at their breakpoints, resulting in increased frequency owing to the selection of breakpoint variants [29]. Although deletions [30, 31] and large inversions [25, 26] in *M. tuberculosis* have been reported, knowledge on the relationship between them remains limited. Given that high-accuracy, HiFi long-read sequencing effectively detects structural variants [32], we successfully assembled the inversion sequence and observed that this rearranged region harbored most deletion and insertion variants. Further investigations are needed to determine the effect of inversion events on genome evolution. Nevertheless, our study provides data that are not yet represented in the WHO catalog and primary data from whole-genome sequencing of *M. tuberculosis* in the DPRK.

## 5. Conclusions

In conclusion, our findings provide comprehensive insights into the genomic features of *M. tuberculosis* PSNK363, along with a description of its complete genome sequence and annotation. The genome of *M. tuberculosis* PSNK363 is approximately 4.4 Mbp with 4079 annotated genes, exhibiting a large-size inversion region. High-accuracy whole-genome sequencing of PSNK363 holds potential for enriching virulence databases and identifying informative loci for drug resistance analysis in *M. tuberculosis* isolates in the DPRK and other countries.

## Figures and Tables

**Table 1:** Comparative genome statistics between PSNK363 and H37Rv.

Isolate	PSNK363	H37Rv
Number of contigs	1	1
Total bases (bp)	4,422,110	4,411,532
N bases	0	0
Max scaffold size (bp)	4,422,110	4,411,532
Min scaffold size (bp)	4,422,110	4,411,532
N50	4,422,110	4,411,532
Protein-coding genes	4079	4018
rRNAs	3	3
tRNAs	53	45

**Table 2:** Number of PSNK363 genes associated with the COG^a^ functional categories.

EggNOG category	Description	Count	Ratio (%)
J	Translation, ribosomal structure, and biogenesis	146	3.5899
A	RNA processing and modification	1	0.0246
K	Transcription	217	5.3356
L	Replication, recombination, and repair	209	5.1389
B	Chromatin structure and dynamics	0	0.0000
D	Cell cycle control, cell division, and chromosome partitioning	43	1.0573
Y	Nuclear structure	0	0.0000
V	Defense mechanisms	49	1.2048
T	Signal transduction mechanisms	99	2.4342
M	Cell wall/membrane/envelope biogenesis	119	2.9260
N	Cell motility	15	0.3688
Z	Cytoskeleton	0	0.0000
W	Extracellular structures	0	0.0000
U	Intracellular trafficking, secretion, and vesicular transport	23	0.5655
O	Posttranslational modification, protein turnover, and chaperones	107	2.6309
C	Energy production and conversion	198	4.8685
G	Carbohydrate transport and metabolism	123	3.0243
E	Amino acid transport and metabolism	203	4.9914
F	Nucleotide transport and metabolism	67	1.6474
H	Coenzyme transport and metabolism	121	2.9752
I	Lipid transport and metabolism	210	5.1635
P	Inorganic ion transport and metabolism	136	3.3440
Q	Secondary metabolites biosynthesis, transport, and catabolism	154	3.7866
R	General function prediction only	715	17.5805
S	Function unknown	1112	27.3420
Total	—	4067	100

Total proteins		4079	
EggNOG DB-matched protein		4006	
a. Single EggNOG		3946	
b. Multi EggNOG		60	
No hit		73	

^a^COG: The clusters of orthologous group [15].

**Table 3:** Classification of gene mutations identified in the PSNK363 assembly based on the WHO catalog^a^.

#CHROM	POS	REF	ALT	Annotation	Gene_Name	Sequence variant	Protein variant	WHO catalog group^a^	
NC_000962.3	620,029	C	T	synonymous_variant	ccsA	c.139C > T	p.Leu47Leu	(4) Not assoc w R-interim	Silent mutation
NC_000962.3	4,038,318	G	A	missense_variant	clpC1	c.2387C > T	p.Pro796Leu	(5) Not assoc w R	
NC_000962.3	2,718,852	T	G	upstream_gene_variant	eis	c.-3520A > C		No in catalog	
NC_000962.3	4,242,643	C	T	synonymous_variant	embC	c.2781C > T	p.Arg927Arg	(5) Not assoc w R	Silent mutation
NC_000962.3	491,433	C	G	synonymous_variant	fgd1	c.651C > G	p.Ala217Ala	No in catalog	
NC_000962.3	7362	G	C	missense_variant	gyrA	c.61G > C	p.Glu21Gln	(5) Not assoc w R	
NC_000962.3	7585	G	C	missense_variant	gyrA	c.284G > C	p.Ser95Thr	(5) Not assoc w R	
NC_000962.3	7892	G	A	synonymous_variant	gyrA	c.591G > A	p.Leu197Leu	(4) Not assoc w R- interim	Silent mutation
NC_000962.3	9304	G	A	missense_variant	gyrA	c.2003G > A	p.Gly668Asp	(5) Not assoc w R	
NC_000962.3	775,639	T	C	missense_variant	mmpL5	c.2842A > G	p.Ile948Val	(5) Not assoc w R	
NC_000962.3	781,395	T	C	upstream_gene_variant	mmpL5	c.-2915A > G		No in catalog	
NC_000962.3	3,625,065	T	G	missense_variant	mtrB	c.1549A > C	p.Met517Leu	(5) Not assoc w R (Rif)	
NC_000962.3	2,170,568	G	C	missense_variant	PPE35	c.45C > G	p.Ile15Met	(5) Not assoc w R	
NC_000962.3	3,065,703	T	C	synonymous_variant	Rv2752c	c.489A > G	p.Gly163Gly	No in catalog	
NC_000962.3	1,917,972	A	G	synonymous_variant	tlyA	c.33A > G	p.Leu11Leu	(5) Not assoc w R	Silent mutation
NC_000962.3	1,854,300	T	C	missense_variant	tsnR	c.695T > C	p.Leu232Pro	(5) Not assoc w R	

*Note:* REF: reference nucleotide. ALT: alternative nucleotide.

^a^Catalog of mutations in *Mycobacterium tuberculosis* complex and their association with drug resistance, 2^nd^ edition.

#CHROM: chromosome number.

**Table 4:** Structural variants of PSNK363 relative to H37Rv.

Ref	Start	End	SV_TYPE^a^	Contig	Start	End	SV_LENGTH	Gene
H37Rv	150,903	150,903	INS	PSNK363	1,764,387	1,764,555	168	PE_PGRS2
H37Rv	334,658	334,658	INS	PSNK363	1,577,828	1,578,554	726	Rv0278c
H37Rv	623,297	623,297	INS	PSNK363	1,289,106	1,289,174	68	PE_PGRS6
H37Rv	669,795	670,966	DEL	PSNK363	1,242,590	1,242,592	1169	Rv0576
H37Rv	742,634	742,635	INS	PSNK363	1,170,352	1,170,886	534	
H37Rv	889,020	890,376	DEL	PSNK363	1,022,499	1,022,499	1356	
H37Rv	1,481,709	1,481,709	INS	PSNK363	429,071	429,967	896	Rv1319c
H37Rv	1,631,544	1,631,620	DEL	PSNK363	279,794	279,794	76	PE_PGRS27
H37Rv	1,989,059	1,996,099	DEL	PSNK363	3,021,890	3,021,890	7040	cut1,wag22,Rv1760,Rv1761c,Rv1762c
H37Rv	1,989,059	1,989,059	INV	PSNK363	3,020,534	3,210,811	190,277	
H37Rv	1,997,463	1,998,658	DEL	PSNK363	3,212,173	3,212,173	1195	Rv1765c
H37Rv	2,061,909	2,062,018	DEL	PSNK363	3,282,976	3,282,976	109	PE_PGRS33
H37Rv	2,219,552	2,219,790	DEL	PSNK363	3,440,348	3,440,348	238	Rv1977
H37Rv	2,231,675	2,232,091	DEL	PSNK363	3,453,589	3,453,589	416	erm(37)
H37Rv	2,268,725	2,268,725	INS	PSNK363	3,490,243	3,491,980	1737	Rv2024c
H37Rv	2,365,414	2,366,769	DEL	PSNK363	3,591,929	3,591,929	1355	
H37Rv	2,430,116	2,431,472	DEL	PSNK363	3,655,272	3,655,272	1356	
H37Rv	2,551,373	2,551,373	INV	PSNK363	1,022,728	3,773,659	2,750,931	
H37Rv	2,634,133	2,634,133	INS	PSNK363	3,856,423	3,858,487	2064	
H37Rv	2,785,972	2,785,972	INS	PSNK363	3,441,704	3,453,589	11,885	
H37Rv	2,785,972	2,785,972	INV	PSNK363	3,440,346	4,008,400	568,054	
H37Rv	2,972,108	2,973,464	DEL	PSNK363	4,194,537	4,194,537	1356	
H37Rv	3,551,229	3,552,585	DEL	PSNK363	2,779,352	2,779,352	1356	
H37Rv	3,710,381	3,711,737	DEL	PSNK363	2,622,922	2,622,922	1356	
H37Rv	3,732,792	3,732,792	INS	PSNK363	2,596,721	2,597,385	664	PPE54
H37Rv	3,847,230	3,847,230	INS	PSNK363	2,479,596	2,479,710	114	PPE59
H37Rv	3,890,778	3,892,134	DEL	PSNK363	2,435,380	2,435,380	1356	
H37Rv	3,949,182	3,949,357	DEL	PSNK363	2,377,434	2,377,434	175	PE_PGRS57

*Note:* INS: insertion, DEL: deletion, INV: inversion.

^a^Types of structural variant identified.

## Data Availability

Raw reads were deposited at the NCBI SRA database under Bioproject PRJNA813637 (https://www.ncbi.nlm.nih.gov/sra/PRJNA813637). The complete assembled genome of PSNK363 is available in NCBI Reference Sequence under accession number NZ_CP143267.1 (https://www.ncbi.nlm.nih.gov/nuccore/NZ_CP143267.1/).
